# Nutritional Mediators of Cellular Decline and Mitochondrial Dysfunction in Older Adults

**DOI:** 10.1093/geroni/igaa057.2758

**Published:** 2020-12-16

**Authors:** Roger Fielding

## Abstract

Aging is the primary risk factor for progressive loss of function, onset of disease, and increased vulnerability to negative health-related outcomes. These clinical manifestations arise from a decline in mitochondrial and metabolic processes considered the hallmarks of aging. Collectively, these changes can be defined as age associated cellular decline (AACD) and are often associated with signs and symptoms such as fatigue, reduced strength and low physical activity. This symposium will explore mechanisms, clinical signs, and emerging nutritional interventions for AACD. Dr. Feige’s presentation will serve as an introduction by highlighting mechanisms underlying functional declines in skeletal muscle with aging. He will discuss the Multi-Ethnic Molecular determinants of Sarcopenia (MEMOSA) study, which found impaired mitochondrial bioenergetic capacity in skeletal muscle of older adults with sarcopenia compared to age-matched controls, and identified mitochondrial function as a key target for intervention. Dr. Guralnik will discuss the connection between cellular changes and clinical manifestations of AACD. He will report on an expert consensus study group which developed an initial framework to identify self-reported symptoms and observable signs of AACD in adults over50 years. Lastly, Dr. Singh will discuss the evidence for nutritional interventions to address sources of AACD, focusing on those targeting mitochondrial dysfunction. Recent research on dietary interventions with urolithin A (an activator of mitophagy) and nicotinamide riboside (an NAD+ booster) will be reviewed. Overall, this symposium will highlight key mechanisms and clinical signs of AACD, and the potential for novel nutrition interventions to support cellular function and healthy aging.

